# MicroRNA-Attenuated Virus Vaccines

**DOI:** 10.3390/ncrna4040025

**Published:** 2018-10-02

**Authors:** Elizabeth J. Fay, Ryan A. Langlois

**Affiliations:** 1 Biochemistry, Molecular Biology, and Biophysics Graduate Program, University of Minnesota, Minneapolis, MN 55455, USA; fayxx084@umn.edu; 2 Center for Immunology, University of Minnesota, Minneapolis, MN 55455, USA; 3 Department of Microbiology and Immunology, University of Minnesota, Minneapolis, MN 55455, USA

**Keywords:** live-attenuated vaccine, RNAi, siRNA

## Abstract

Live-attenuated vaccines are the most effective way to establish robust, long-lasting immunity against viruses. However, the possibility of reversion to wild type replication and pathogenicity raises concerns over the safety of these vaccines. The use of host-derived microRNAs (miRNAs) to attenuate viruses has been accomplished in an array of biological contexts. The broad assortment of effective tissue- and species-specific miRNAs, and the ability to target a virus with multiple miRNAs, allow for targeting to be tailored to the virus of interest. While escape is always a concern, effective strategies have been developed to improve the safety and stability of miRNA-attenuated viruses. In this review, we discuss the various approaches that have been used to engineer miRNA-attenuated viruses, the steps that have been taken to improve their safety, and the potential use of these viruses as vaccines.

## 1. Introduction

MicroRNAs (miRNAs) are short RNAs that bind with partial complementarity to mRNAs to inhibit translation of their target. miRNAs are transcribed by Pol II and form a secondary hairpin structure that is initially processed in the nucleus by the RNase III enzyme Drosha. Following nuclear export, additional processing is completed by a second RNase III enzyme, Dicer. The miRNA is then loaded into the multi-protein RNA-induced silencing complex (RISC), which mediates mRNA decay and translational inhibition. miRNA bind to target mRNAs through the seed sequence, nucleotides 2–8 on the 5’ end of the miRNA, resulting in translation repression of ~2 fold. However, perfect complementarity can result in target mRNA cleavage and significant enhancement of repression. In rare cases of binding with perfect complementarity between miRNA-mRNA sequences, mRNA cleavage will be induced. In eukaryotes, miRNAs are essential regulators of mRNA expression during development and fine tune translation to control other diverse cellular processes [1].

Plants and invertebrates use miRNAs for gene regulation, but also have a separate, partially overlapping antiviral defense mechanism, RNA interference (RNAi). Similar to miRNAs, small interfering RNA (siRNA) processing is carried out by Dicer and siRNAs are loaded into RISC, although in many organisms, there are generally separate versions of these proteins for each pathway. In these species, the processing of long, double-stranded viral RNA and miRNA processing are carried out by separate Dicer proteins, but share components of other steps of the RNAi pathway [2,3]. As an antiviral mechanism, silencing through RNAi is achieved by producing virus-derived siRNAs that bind with perfect complementarity to the target viral sequence. While small virus-derived RNAs have been identified in mammalian cells following infection [4], siRNAs are not the primary antiviral mechanism in mammals [5,6]. However, while not naturally antiviral, the miRNA pathway can be experimentally coopted to repress virus replication. Importantly, miRNA and siRNA function through the same mechanisms in mammalian cells, suggesting that miRNAs could be exploited to be antiviral [7]. This has been achieved by inserting perfectly complementary target sites for the miRNA into the viral gene of interest, effectively turning the miRNA into an siRNA. Tissue- and species-specific miRNAs have been exploited to control virus replication in a myriad of biological contexts, including to improve the safety of oncolytic viruses by attenuating the virus in non-tumor tissues [8,9,10,11] and to attain tissue-specific expression of virus-derived transgenes or to limit the immune response against the transgene for gene therapy [12,13,14]. These studies have demonstrated the ability of engineered miRNA targeting to control virus replication in vitro and in vivo.

Vaccines have been highly successful at limiting viral infections and have led to the elimination of smallpox from the human population [15]. There are four main classes of viral vaccines: killed, subunit, mRNA, and live-attenuated. Live-attenuated vaccines have several advantages, including providing more robust, long-lasting immunity compared to inactivated vaccines [16]. Viruses can be attenuated through several different mechanisms, including altering the temperature of the optimal polymerase function and deleting or mutating viral immune antagonists. However, some effective attenuation strategies result in poor immunogenicity, limiting their use as a vaccine. Mechanisms of attenuation may not be identical across viral species. For example, the mechanism of temperature sensitivity will vary from virus to virus. Additionally, safety is a major concern when developing any live-attenuated vaccine, where reversion to wild type replication could be devastating [16]. miRNAs have been used as a platform to develop live-attenuated vaccines for DNA and both positive and negative sense RNA viruses by exploiting host endogenous miRNAs. The broad array of cell- and species-specific miRNAs, as well as the ability to target using multiple miRNAs, allow for customizable attenuation for different viruses. A major advantage of this strategy is that this provides a known mechanism of attenuation, which can be applied across a diverse range of viruses. In this review, we will discuss the approaches for generating miRNA-targeted viruses and several strategies for improving the safety and efficacy of miRNA-attenuated vaccines.

## 2. MicroRNA-Attenuated Vaccines

### 2.1. Mechanisms of MicroRNA Targeting of Viruses

Endogenous miRNA targeting primarily occurs at the three prime untranslated region (3’ UTR) of host mRNA transcripts [17]. However, many viral RNAs contain short 3’ UTRs, potentially as an evolutionary mechanism to evade miRNA regulation. There are two ways that viruses can be engineered to be sensitive to miRNAs: through the generation of silent mutations in the open reading frame to be complementary to the cognate miRNA or by inserting complete target sites into the endogenous or engineered UTRs. Some locations in the viral genome may not be amenable to miRNA machinery access due to the secondary RNA structure or binding of viral proteins, and therefore cannot be used to attenuate virus replication [18]. West Nile virus has been engineered to encode miRNA target sites between RNA secondary structures in the 3’ UTR to allow miRNA access and to prevent disruption of the important RNA structures [19]. These results demonstrate that care must be taken in choosing genomic sites for miRNA targeting. In addition to the location within a gene, engineered miRNA-mediated repression can be affected by the choice of viral gene being silenced. Targeting an essential gene can completely disrupt virus replication, while the targeting of a non-essential gene can prevent pathology but allow for the expression of some viral products, which may be necessary to produce a robust adaptive immune response. Reversion to virulence is a concern for all live-attenuated vaccines, including miRNA-targeted vaccines. Escape from miRNA targeting, as well as strategies to mitigate this risk, are discussed in a later section of this review.

Some DNA viruses naturally encode miRNAs to regulate the viral replication life cycle and control host gene expression [20,21,22]. RNA viruses have thus far been found to be devoid of this regulatory mechanism (with the notable exception of some retroviruses [23,24,25,26]). However, both positive and negative sense RNA viruses can be engineered to express a functional miRNA [27,28,29,30,31], which can be further exploited to generate self-attenuating viruses. Inserting an artificial miRNA with perfect sequence complementarity to nucleoprotein as an intron in the nonstructural gene segment created a self-attenuating influenza virus [32]. This strategy resulted in highly attenuated replication in vivo, even in the absence of a type I interferon response. Work from another group demonstrated that this self-attenuating virus could be used as a protective influenza virus vaccine [33]. However, as discussed in more detail below, the major drawback to this strategy is rapid escape from attenuation through loss of the artificial miRNA [32].

### 2.2. Species-, Tissue-, and Cell-Specific MicroRNA Targeting of Viruses

In a landmark study, Landgraf et al. developed an atlas of miRNA expression in a variety of cell types and tissues from mice and humans [34]. This study demonstrated that while the majority of miRNAs are broadly expressed, there are several whose expression is restricted to particular cell types, lineages, or tissues [34]. However, different tissue- or host-specific miRNAs may not repress viral gene expression equally, and the level of attenuation is of critical importance when designing a miRNA-attenuated vaccine. For example, three different central nervous system (CNS)-specific miRNAs effectively attenuated a targeted flavivirus in vitro, but only two were able to prevent pathogenesis and death in vivo [35]. This suggests that the presence of a miRNA alone cannot always predict target efficacy. One potential explanation for this is that endogenous targets for a miRNA can act as sponges, reducing the functional amount of a miRNA within a cell. Furthermore, it has been suggested that 100–1000 miRNAs need to be present in the cell for at least 6 h after infection to repress engineered miRNA sensitive virus replication [32]. Using a fluorescence-based screening approach, Mullokandov et al. were able to determine the functional repression capacity of endogenous miRNAs in a variety of cell types [36], and this platform can be used to screen for effective miRNAs for attenuated vaccines. One potential area of concern is altered miRNA expression during virus infection. Many acute RNA viruses replicate rapidly and the life cycle may be shorter than the time needed to upregulate sufficient quantities of antiviral miRNAs [37]. However, infections with a number of viruses have demonstrated changes in the overall pattern of miRNA expression. Therefore, infection-specific changes in miRNA expression should be considered, particularly for viruses with the capacity for latency. Overall, the capacity of a miRNA to repress targeted virus replication is difficult to predict based on small RNA abundance alone. Consideration of other factors and experimental validation in the relevant cells and systems are critical.

One strategy for the generation of a live-attenuated vaccine using miRNAs is to target the virus only in cells or tissues that underlie pathogenesis for that infection. Targeting tick-borne encephalitis virus or dengue virus using a CNS-specific miRNA limited neuropathogenesis in mice, while preserving immunogenicity [38]. Importantly, neutralizing antibodies could be generated following the inoculation of non-human primates with the targeted virus [38]. Similar results were obtained for Japanese encephalitis virus [39] and West Nile virus [19]. Muscle-specific miRNA-targeted Coxsackie B virus displayed reduced cardiopathology and generated a strong protective immune response [40]. In these examples, viral infection could still occur peripherally, including in antigen presenting cells, which potentially allows for the increased generation of adaptive immune responses. Influenza virus has a broad tropism and is able to replicate in both epithelial and immune cells [41]. Using a hematopoietic-specific miRNA to block influenza virus replication in immune cells still resulted in robust activation of CD8 T cells [42]. Because antigen presenting cells could not be directly infected, these data suggest that exogenous antigen acquisition and cross-presentation are sufficient to generate anti-influenza cell-mediated immunity. These studies suggest that blocking pathogenic replication, but allowing replication in other cells, is sufficient to generate robust antiviral immunity.

**Figure 1:**
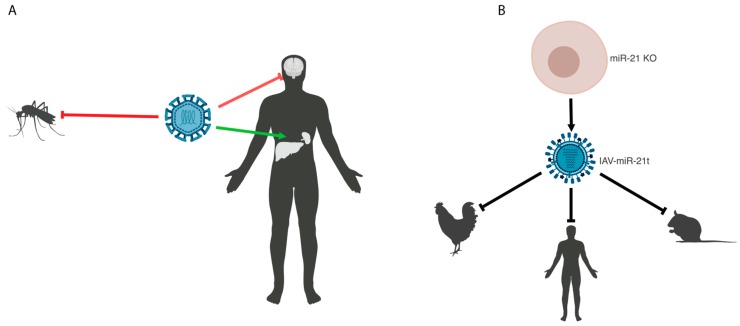
Tissue- and species-specific microRNA-attenuated viruses. (**A**) Model of tissue-specific attenuation of a flavivirus combined with attenuation in an insect vector. (**B**) Model depicting generation of a miRNA-attenuated influenza A virus in miRNA knock out cells to generate a species-universal attenuated vaccine. Created with BioRender (Toronto, ON, Canada).

Many viruses that cause disease in humans also replicate in or are transmitted through non-human species. Continued replication or recombination of miRNA-attenuated vaccine strains in these species could result in the loss of miRNA target sites and reversion to a wild type strain, which could lead to the spread of the pathogenic virus to other hosts. It is therefore critical that vaccines that are attenuated in humans are also blunted in these zoonotic reservoirs. Attenuation of viruses using multiple species-specific miRNAs can improve the safety of live-attenuated vaccines. Mosquito-specific and tick-specific miRNAs have been used in combination with CNS-specific miRNAs to attenuate flaviviruses in the natural vectors and prevent the escape of vaccine strains [19,35,43] (Figure 1A). While there is a diverse array of tissue- and host-specific miRNAs, patterns of miRNA expression are not always perfectly tailored to the desired application. To circumvent this, Waring et al. eliminated a eukaryotic ubiquitously expressed miRNA, miR-21, from MDCK cells to allow for the growth of a targeted influenza virus [44]. This virus was attenuated in eukaryotic hosts, including avian and human cells and in mice [44] (Figure 1B). This approach allowed for a species universal attenuated vaccine, which provided robust protection upon lethal challenge in mice. This study also provides a platform for molecular biocontainment to prevent the spread of engineered influenza virus into human or zoonotic reservoirs, while still allowing for experimental analyses in cell lines lacking the cognate miRNA.

As discussed above, miRNA targeting can be used to restrict a virus such that it can only replicate in the necessary viral amplification platform. Influenza viruses, including those used for vaccines, are grown to a high titer in embryonated chicken eggs. To generate influenza virus vaccines that could be grown in eggs but would be attenuated in mammals, Perez et al. screened the small miRNAs in avian and mammalian cells and found several that were absent in eggs but present in mammals. Using one of these, miR-93, they generated a vaccine that could replicate in eggs but was attenuated in mice [45]. Importantly, this vaccine provided robust protection upon challenge with lethal influenza strains. Species-specific attenuation can also be used to enhance safety when working with pathogenic viruses in the laboratory through molecular biocontainment. miR-192 is present in human lung, but absent from ferret lung. Targeting the influenza virus with miR-192 allowed for experimentation in ferrets without the concern for human infection [46]. Together, these studies demonstrate the plasticity in using cell- and species-specific miRNAs to control virus replication for the generation of live-attenuated vaccines.

### 2.3. Immunogenicity of MicroRNA-Attenuated Viruses

One concern with any live attenuated vaccine strategy is that replication will be reduced to a point where the immune response to the virus is severely compromised, resulting in insufficient protection from secondary infection. It is therefore important to characterize the innate and adaptive immune responses to miRNA-attenuated viruses. Benitez et al. found that a miRNA-targeted influenza virus induced the expression of a myriad of interferon-stimulated genes in mice, suggesting that the robust attenuation of the virus still allowed activation of the innate immune response [32]. In another influenza study, a miRNA-attenuated vaccine generated high anti-influenza A virus antibody titers in mice, despite the lack of detectable virus replication, and an equivalent dose of UV-killed virus failed to induce protection [44]. Similar results have been shown for attenuated flavivirus and enterovirus, where miRNA-mediated attenuation prevented disease, but still allowed for the generation of a protective adaptive immune response [38,39,40,44]. In the event that a virus is attenuated to the point where it is poorly immunogenic, there are strategies to improve immunogenicity. For example, increasing the starting inoculum can raise antibody titers while still preventing pathogenesis [19]. miRNA-mediated attenuation could potentially allow for increased inoculums compared to other live attenuated vaccine strategies as a strategy to increase immunogenicity. As has been shown for influenza virus, targeting different genes can result in differential attenuation, and targeting multiple genes can improve attenuation over targeting single genes [44]. If targeting an essential gene fails to induce sufficient immunity, this strategy of customizable attenuation using miRNAs can be employed to achieve appropriate levels of replication to generate an immune response without causing disease. Altogether, these studies demonstrate that miRNA-attenuation can prevent pathogenesis of the virus while still initiating an immune response that results in protection from secondary infections.

### 2.4. Escape from MicroRNA Targeting

Replication in the presence of the cognate miRNA applies selective pressure for the potential mutation or complete loss of miRNA target sites. Reduced targeting is a major concern when designing miRNA-attenuated vaccines. A single let-7 target site in poliovirus can accumulate escape mutations as early as 24 h post infection, restoring the full replicative potential of the virus [47]. Additionally, a single nucleotide mutation in a miRNA target site can restore the neurovirulence of a targeted flavivirus [38]. One way to mitigate escape is to engineer multiple target sites into the virus. Targeting only the 3’ UTR of Langat virus resulted in escape after multiple replication cycles due to the deletion of miRNA target sites, but when multiple genomic loci were targeted, miRNA target sites were retained and the virus did not escape [35]. Another study using influenza virus demonstrated that even reducing perfect complementarity from 20 base pairs to 16 base pairs did not result in escape when two target sites were inserted [32], further illustrating the benefit of multiple sites. Additionally, targeting multiple segments of influenza virus increased attenuation from segments that resulted in poor attenuation individually [44], demonstrating the power of combinatorial targeting. In addition to using multiple target sites for the same miRNA, enhanced viral repression can be achieved using multiple different miRNAs, and this strategy can be more effective at attenuating than multiple sites for the same miRNA [43]. However, studies still need to be done to determine how the order of miRNA target sites impacts the efficiency of each individual site within a multi-targeting cassette. A self-attenuating influenza virus engineered to express an artificial miRNA targeting its own genome also demonstrated escape. However, this occurred through deletions in the hairpin, not in the miRNA target site [32], indicating that endogenous target sites are not readily amenable to escape mutations, likely because viral genomic sequences are highly conserved. This is in contrast to engineered target sites where, for example, Dengue virus completely lost miRNA target sequences after infection in vivo [48]. While multiple target sites can improve safety and are likely necessary for designing safe miRNA-attenuated vaccines, additional strategies may be required to overcome viral escape through the loss of target sites.

**Figure 2:**
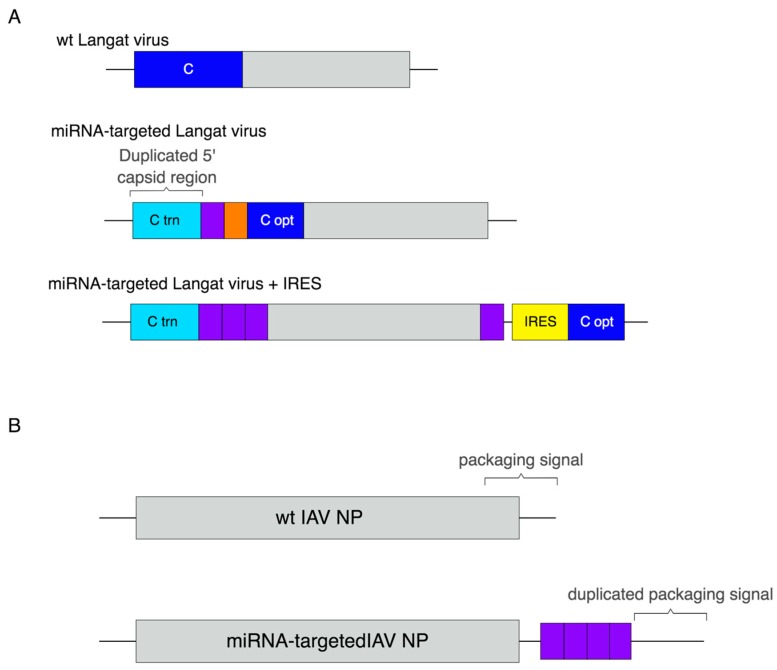
Altered viral genome structures to improve miRNA targeting. (**A**) Model of the wild type Langat virus genome, highlighting the capsid gene (top), the miRNA-targeted (purple) duplicated 5’ region followed by a 2A site (orange) to allow for expression of the codon-optimized capsid protein (middle), and the insertion of an IRES to regulate the expression of the capsid protein (bottom). (**B**) The wild type influenza virus NP gene (top) and the miRNA-targeted (purple) NP gene with a duplicated packaging signal (bottom). Created with BioRender (Toronto, ON, Canada).

Several alterations in the viral genome have improved the stability and efficacy of miRNA-mediated attenuation of flaviviruses and other viruses. One way that this has been achieved is by targeting only the artificially duplicated 5’ regulatory region of the Langat virus capsid RNA, leaving the optimized coding region intact. This method of targeting was more effective than targeting the viral 3’ UTR. Additionally, insertions in the duplicated 5’ region mitigated the risk of homologous recombination between the duplicated region and the coding region, improving the stability of the miRNA target sites [49]. This group also combined this strategy with insertion of the encephalomyocarditis virus internal ribosomal entry site (IRES) upstream of the capsid gene. This reduced the abundance of the capsid protein and caused general attenuation of the virus. Combining IRES-mediated attenuation with miRNA targeting improved the stability and effectiveness of miRNA attenuation compared to miRNA targeting alone [50]. This clever strategy also mitigated the risk of mutation in insect vectors, as RNA translation from the encephalomyocarditis virus IRES is inhibited in insect cells and the virus was therefore unable to replicate [51] (Figure 2A). While targeting the 3’ UTR is effective, the secondary RNA structure in this region may block miRNA targeting. Additionally, some viral UTRs contain packaging signals that need to remain intact to successfully incorporate viral genomes into the virion. Duplicating the 3’ UTR, which was done to insert miRNA target sites into influenza virus genes [42,44,46], retains the necessary packaging information and may allow for a linear single stranded targeting region, enhancing the efficiency of repression (Figure 2B). Dengue virus completely lost miRNA target sequences after infection in vivo [48], suggesting that genomic alterations may be necessary to produce a safe, stable miRNA-attenuated Dengue virus vaccine. While the strategies described above have been successful, genomic changes may need to be tailored for use in other viruses. Together, these data illustrate that increasing the number of target sites and engineering alterations to the virus genome can improve miRNA-mediated repression and mitigate the risk of escape.

### 2.5. Viral Suppressors of MicroRNAs

RNAi is the primary antiviral defense mechanism in many non-vertebrate species. Therefore, viruses that infect these hosts have evolved mechanisms to evade this response. Most plant viruses encode a protein that inhibits some component of the antiviral RNAi pathway [2]. Similarly, many invertebrate viruses encode a suppressor of RNAi, including suppressors of Dicer and RISC proteins [52,53,54,55,56]. Viruses that infect both invertebrates and mammals may possess these immune evasion mechanisms, and because the miRNA and RNAi pathways share many of the same components, virus proteins that target common elements would impact miRNA-mediated attenuation. Strikingly, arboviruses, which infect arthropods and mammals, do not appear to express viral suppressors of RNAi, despite being susceptible to the arthropod antiviral RNAi response [57]. However, West Nile virus may evade RNAi by positive selection for point mutations that prevent targeting [58]. Several DNA viruses are capable of suppressing miRNAs. For example, the poxvirus protein VP55, an essential component of the viral polyA polymerase, can additionally non-specifically polyadenylate miRNAs, resulting in their eventual destruction [59]. Adenoviruses inhibit both the nuclear export of miRNAs, as well as Dicer function [60]. There are several reports of influenza NS1 blocking RNAi. However we, and others, have shown that miRNAs can attenuate targeted influenza, even in the presence of increased NS1 expression [32,42,45,46,61,62]. These studies indicate that not all viruses can be candidates for miRNA-mediated attenuation unless steps are taken to cripple miRNA suppressive viral genes.

While mammalian viruses do not appear to express suppressors of RNAi, there may be mechanisms that indirectly inhibit the miRNA targeting of viruses. Induction of the interferon response in the presence of virus replication has been shown to inhibit miRNA function via poly-ADP-ribosylation of Ago2, an essential component of RISC [63]. This could reduce miRNA-mediated attenuation during vaccination/infection and provide a window for escape. The inhibition of RNAi by the interferon response suggests that these are not compatible systems and that miRNAs are not an effective intrinsic antiviral strategy in mammals. Furthermore, the absence of miRNAs from a cell did not hinder the replication of a variety of viruses, suggesting that miRNAs are not a potent natural antiviral defense mechanism for the host [64,65]. The lack of a mammalian antiviral RNAi system suggests that mammalian viruses would not need to evolve suppressors of this pathway, further supporting the ability to experimentally coopt miRNA targeting for safe and effective virus attenuation.

## 3. Conclusions

miRNA-mediated attenuation has been achieved for many viruses in many hosts. Several strategies have been employed that have improved the safety, stability, and efficacy of miRNA-mediated attenuation for the purpose of generating live-attenuated vaccines. The utility of miRNA targeting of viruses extends beyond vaccines and can be used for gene therapy and oncolytic virotherapy, and this strategy could be extended to non-viral pathogens by using viral vectors. Overall, miRNA targeting is a promising platform for developing safe, effective vaccines and provides increased plasticity over traditional live-attenuated vaccine strategies.

## References

[1] Bartel D.P. (2018). Metazoan MicroRNAs. Cell.

[2] Szittya G., Burgyan J., Cullen B. (2013). RNA interference-mediated intrinsic antiviral immunity in plants. Intrinsic Immunity.

[3] Nayak A., Tassetto M., Kunitomi M., Andino R., Cullen B. (2013). RNA interference-mediated intrinsic antiviral immunity in invertebrates. Intrinsic Immunity.

[4] Parameswaran P., Sklan E., Wilkins C., Burgon T., Samuel M.A., Lu R., Ansel K.M., Heissmeyer V., Einav S., Jackson W. (2010). Six RNA viruses and forty-one hosts: Viral small RNAs and modulation of small RNA repertoires in vertebrate and invertebrate systems. PLoS Pathog..

[5] tenOever B.R. (2017). Questioning antiviral RNAi in mammals. Nat. Microbiol..

[6] Cullen B.R., Cherry S., tenOever B.R. (2013). Is RNA interference a physiologically relevant innate antiviral immune response in mammals?. Cell Host Microbe.

[7] Zeng Y., Yi R., Cullen B.R. (2003). MicroRNAs and small interfering RNAs can inhibit mRNA expression by similar mechanisms. Proc. Natl. Acad. Sci. USA.

[8] Kelly E.J., Hadac E.M., Greiner S., Russell S.J. (2008). Engineering microRNA responsiveness to decrease virus pathogenicity. Nat. Med..

[9] Kelly E.J., Nace R., Barber G.N., Russell S.J. (2010). Attenuation of vesicular stomatitis virus encephalitis through microRNA targeting. J. Virol..

[10] Ruiz A.J., Hadac E.M., Nace R.A., Russell S.J. (2016). MicroRNA-detargeted mengovirus for oncolytic virotherapy. J. Virol..

[11] Edge R.E., Falls T.J., Brown C.W., Lichty B.D., Atkins H., Bell J.C. (2008). A let-7 MicroRNA-sensitive vesicular stomatitis virus demonstrates tumor-specific replication. Mol. Ther..

[12] Brown B.D., Venneri M.A., Zingale A., Sergi Sergi L., Naldini L. (2006). Endogenous microRNA regulation suppresses transgene expression in hematopoietic lineages and enables stable gene transfer. Nat. Med..

[13] Suzuki T., Sakurai F., Nakamura S., Kouyama E., Kawabata K., Kondoh M., Yagi K., Mizuguchi H. (2008). miR-122a-regulated expression of a suicide gene prevents hepatotoxicity without altering antitumor effects in suicide gene therapy. Mol. Ther..

[14] Xie J., Xie Q., Zhang H., Ameres S.L., Hung J.H., Su Q., He R., Mu X., Seher Ahmed S., Park S. (2011). MicroRNA-regulated, systemically delivered rAAV9: A step closer to CNS-restricted transgene expression. Mol. Ther..

[15] Henderson D.A. (1980). Smallpox eradication. Public Health Rep..

[16] Lauring A.S., Jones J.O., Andino R. (2010). Rationalizing the development of live attenuated virus vaccines. Nat. Biotechnol..

[17] Bartel D.P. (2009). MicroRNAs: Target recognition and regulatory functions. Cell.

[18] Gismondi M.I., Ortiz X.P., Curra A.P., Asurmendi S., Taboga O. (2014). Artificial microRNAs as antiviral strategy to FMDV: Structural implications of target selection. J. Virol. Methods.

[19] Brostoff T., Pesavento P.A., Barker C.M., Kenney J.L., Dietrich E.A., Duggal N.K., Bosco-Lauth A.M., Brault A.C. (2016). MicroRNA reduction of neuronal West Nile virus replication attenuates and affords a protective immune response in mice. Vaccine.

[20] Sullivan C.S., Grundhoff A.T., Tevethia S., Pipas J.M., Ganem D. (2005). SV40-encoded microRNAs regulate viral gene expression and reduce susceptibility to cytotoxic T cells. Nature.

[21] Umbach J.L., Kramer M.F., Jurak I., Karnowski H.W., Coen D.M., Cullen B.R. (2008). MicroRNAs expressed by herpes simplex virus 1 during latent infection regulate viral mRNAs. Nature.

[22] Gottwein E., Mukherjee N., Sachse C., Frenzel C., Majoros W.H., Chi J.T., Braich R., Manoharan M., Soutschek J., Ohler U. (2007). A viral microRNA functions as an orthologue of cellular miR-155. Nature.

[23] Kincaid R.P., Burke J.M., Sullivan C.S. (2012). RNA virus microRNA that mimics a B-cell oncomiR. Proc. Natl. Acad. Sci. USA.

[24] Burke J.M., Bass C.R., Kincaid R.P., Sullivan C.S. (2014). Identification of tri-phosphatase activity in the biogenesis of retroviral microRNAs and RNAP III-generated shRNAs. Nucleic Acids Res..

[25] Whisnant A.W., Kehl T., Bao Q., Materniak M., Kuzmak J., Lochelt M., Cullen B.R. (2014). Identification of novel, highly expressed retroviral microRNAs in cells infected by bovine foamy virus. J. Virol..

[26] Kincaid R.P., Chen Y., Cox J.E., Rethwilm A., Sullivan C.S. (2014). Noncanonical microRNA (miRNA) biogenesis gives rise to retroviral mimics of lymphoproliferative and immunosuppressive host miRNAs. mBio.

[27] Shapiro J.S., Langlois R.A., Pham A.M., tenOever B.R. (2012). Evidence for a cytoplasmic microprocessor of pri-miRNAs. RNA.

[28] Langlois R.A., Shapiro J.S., Pham A.M., tenOever B.R. (2012). In vivo delivery of cytoplasmic RNA virus-derived miRNAs. Mol. Ther..

[29] Rouha H., Thurner C., Mandl C.W. (2010). Functional microRNA generated from a cytoplasmic RNA virus. Nucleic Acids Res..

[30] Shapiro J.S., Varble A., Pham A.M., tenOever B.R. (2010). Noncanonical cytoplasmic processing of viral microRNAs. RNA.

[31] Varble A., Chua M.A., Perez J.T., Manicassamy B., Garcia-Sastre A., tenOever B.R. (2010). Engineered RNA viral synthesis of microRNAs. Proc. Natl. Acad. Sci. USA.

[32] Benitez A.A., Spanko L.A., Bouhaddou M., Sachs D., tenOever B.R. (2015). Engineered Mammalian RNAi Can Elicit Antiviral Protection that Negates the Requirement for the Interferon Response. Cell Rep..

[33] Li J., Arevalo M.T., Diaz-Arevalo D., Chen Y., Choi J.G., Zeng M. (2015). Generation of a safe and effective live viral vaccine by virus self-attenuation using species-specific artificial microRNA. J. Control. Release.

[34] Landgraf P., Rusu M., Sheridan R., Sewer A., Iovino N., Aravin A., Pfeffer S., Rice A., Kamphorst A.O., Landthaler M. (2007). A mammalian microRNA expression atlas based on small RNA library sequencing. Cell.

[35] Tsetsarkin K.A., Liu G., Kenney H., Hermance M., Thangamani S., Pletnev A.G. (2016). Concurrent micro-RNA mediated silencing of tick-borne flavivirus replication in tick vector and in the brain of vertebrate host. Sci. Rep..

[36] Mullokandov G., Baccarini A., Ruzo A., Jayaprakash A.D., Tung N., Israelow B., Evans M.J., Sachidanandam R., Brown B.D. (2012). High-throughput assessment of microRNA activity and function using microRNA sensor and decoy libraries. Nat. Methods.

[37] Tenoever B.R. (2013). RNA viruses and the host microRNA machinery. Nat. Rev. Microbiol..

[38] Heiss B.L., Maximova O.A., Pletnev A.G. (2011). Insertion of microRNA targets into the flavivirus genome alters its highly neurovirulent phenotype. J. Virol..

[39] Yen L.C., Lin Y.L., Sung H.H., Liao J.T., Tsao C.H., Su C.M., Lin C.K., Liao C.L. (2013). Neurovirulent flavivirus can be attenuated in mice by incorporation of neuron-specific microRNA recognition elements into viral genome. Vaccine.

[40] He F., Yao H., Wang J., Xiao Z., Xin L., Liu Z., Ma X., Sun J., Jin Q., Liu Z. (2015). Coxsackievirus B3 engineered to contain microRNA targets for muscle-specific microRNAs displays attenuated cardiotropic virulence in mice. J. Virol..

[41] Fiege J.K., Langlois R.A. (2015). Investigating influenza a virus infection: Tools to track infection and limit tropism. J. Virol..

[42] Langlois R.A., Varble A., Chua M.A., Garcia-Sastre A., tenOever B.R. (2012). Hematopoietic-specific targeting of influenza A virus reveals replication requirements for induction of antiviral immune responses. Proc. Natl. Acad. Sci. USA.

[43] Tsetsarkin K.A., Liu G., Kenney H., Bustos-Arriaga J., Hanson C.T., Whitehead S.S., Pletnev A.G. (2015). Dual miRNA targeting restricts host range and attenuates neurovirulence of flaviviruses. PLoS Pathog..

[44] Waring B.M., Sjaastad L.E., Fiege J.K., Fay E.J., Reyes I., Moriarity B., Langlois R.A. (2017). MicroRNA-based attenuation of influenza virus across susceptible hosts. J. Virol..

[45] Perez J.T., Pham A.M., Lorini M.H., Chua M.A., Steel J., tenOever B.R. (2009). MicroRNA-mediated species-specific attenuation of influenza A virus. Nat. Biotechnol..

[46] Langlois R.A., Albrecht R.A., Kimble B., Sutton T., Shapiro J.S., Finch C., Angel M., Chua M.A., Gonzalez-Reiche A.S., Xu K. (2013). MicroRNA-based strategy to mitigate the risk of gain-of-function influenza studies. Nat. Biotechnol..

[47] Vignuzzi M., Wendt E., Andino R. (2008). Engineering attenuated virus vaccines by controlling replication fidelity. Nat. Med..

[48] Pham A.M., Langlois R.A., Tenoever B.R. (2012). Replication in cells of hematopoietic origin is necessary for Dengue virus dissemination. PLoS Pathog..

[49] Tsetsarkin K.A., Liu G., Shen K., Pletnev A.G. (2016). Kissing-loop interaction between 5′ and 3′ ends of tick-borne Langat virus genome ‘bridges the gap’ between mosquito- and tick-borne flaviviruses in mechanisms of viral RNA cyclization: Applications for virus attenuation and vaccine development. Nucleic Acids Res..

[50] Tsetsarkin K.A., Liu G., Volkova E., Pletnev A.G. (2017). Synergistic Internal Ribosome Entry Site/MicroRNA-Based Approach for Flavivirus Attenuation and Live Vaccine Development. mBio.

[51] Woolaway K.E., Lazaridis K., Belsham G.J., Carter M.J., Roberts L.O. (2001). The 5′ untranslated region of Rhopalosiphum padi virus contains an internal ribosome entry site which functions efficiently in mammalian, plant, and insect translation systems. J. Virol..

[52] Chao J.A., Lee J.H., Chapados B.R., Debler E.W., Schneemann A., Williamson J.R. (2005). Dual modes of RNA-silencing suppression by Flock House virus protein B2. Nat. Struct. Mol. Biol..

[53] Van Rij R.P., Saleh M.C., Berry B., Foo C., Houk A., Antoniewski C., Andino R. (2006). The RNA silencing endonuclease Argonaute 2 mediates specific antiviral immunity in Drosophila melanogaster. Genes Dev..

[54] Nayak A., Berry B., Tassetto M., Kunitomi M., Acevedo A., Deng C., Krutchinsky A., Gross J., Antoniewski C., Andino R. (2010). Cricket paralysis virus antagonizes Argonaute 2 to modulate antiviral defense in Drosophila. Nat. Struct. Mol. Biol..

[55] Qi N., Zhang L., Qiu Y., Wang Z., Si J., Liu Y., Xiang X., Xie J., Qin C.F., Zhou X. (2012). Targeting of dicer-2 and RNA by a viral RNA silencing suppressor in Drosophila cells. J. Virol..

[56] van Mierlo J.T., Bronkhorst A.W., Overheul G.J., Sadanandan S.A., Ekstrom J.O., Heestermans M., Hultmark D., Antoniewski C., van Rij R.P. (2012). Convergent evolution of argonaute-2 slicer antagonism in two distinct insect RNA viruses. PLoS Pathog..

[57] Blair C.D. (2011). Mosquito RNAi is the major innate immune pathway controlling arbovirus infection and transmission. Future Microbiol..

[58] Brackney D.E., Beane J.E., Ebel G.D. (2009). RNAi targeting of West Nile virus in mosquito midguts promotes virus diversification. PLoS Pathog..

[59] Backes S., Shapiro J.S., Sabin L.R., Pham A.M., Reyes I., Moss B., Cherry S., tenOever B.R. (2012). Degradation of host microRNAs by poxvirus poly(A) polymerase reveals terminal RNA methylation as a protective antiviral mechanism. Cell Host Microbe.

[60] Lu S., Cullen B.R. (2004). Adenovirus VA1 noncoding RNA can inhibit small interfering RNA and MicroRNA biogenesis. J. Virol..

[61] Li H., Bradley K.C., Long J.S., Frise R., Ashcroft J.W., Hartgroves L.C., Shelton H., Makris S., Johansson C., Cao B. (2018). Internal genes of a highly pathogenic H5N1 influenza virus determine high viral replication in myeloid cells and severe outcome of infection in mice. PLoS Pathog..

[62] Tundup S., Kandasamy M., Perez J.T., Mena N., Steel J., Nagy T., Albrecht R.A., Manicassamy B. (2017). Endothelial cell tropism is a determinant of H5N1 pathogenesis in mammalian species. PLoS Pathog..

[63] Seo G.J., Kincaid R.P., Phanaksri T., Burke J.M., Pare J.M., Cox J.E., Hsiang T.Y., Krug R.M., Sullivan C.S. (2013). Reciprocal inhibition between intracellular antiviral signaling and the RNAi machinery in mammalian cells. Cell Host Microbe.

[64] Bogerd H.P., Whisnant A.W., Kennedy E.M., Flores O., Cullen B.R. (2014). Derivation and characterization of Dicer- and microRNA-deficient human cells. RNA.

[65] Backes S., Langlois R.A., Schmid S., Varble A., Shim J.V., Sachs D., Tenoever B.R. (2014). The Mammalian response to virus infection is independent of small RNA silencing. Cell Rep..

